# Impacts of Adaptive Statistical Iterative Reconstruction-V and Deep Learning Image Reconstruction Algorithms on Robustness of CT Radiomics Features: Opportunity for Minimizing Radiomics Variability Among Scans of Different Dose Levels

**DOI:** 10.1007/s10278-023-00901-1

**Published:** 2024-01-29

**Authors:** Jingyu Zhong, Zhiyuan Wu, Lingyun Wang, Yong Chen, Yihan Xia, Lan Wang, Jianying Li, Wei Lu, Xiaomeng Shi, Jianxing Feng, Haipeng Dong, Huan Zhang, Weiwu Yao

**Affiliations:** 1 Department of Imaging, Tongren Hospital, Shanghai Jiao Tong University School of Medicine, Shanghai, 200336 China; 2 Department of Radiology, Ruijin Hospital, Shanghai Jiao Tong University School of Medicine, Shanghai, 200025 China; 3 Computed Tomography Research Center, GE Healthcare, Beijing, 100176 China; 4 Computed Tomography Research Center, GE Healthcare, Shanghai, 201203 China; 5 Department of Materials, Imperial College London, South Kensington Campus, London, SW7 2AZ UK; 6 Haohua Technology Co., Ltd., Shanghai, 201100 China

**Keywords:** Deep learning, Multidetector computed tomography, Reproducibility of results, Image enhancement, Image reconstruction

## Abstract

This study aims to investigate the influence of adaptive statistical iterative reconstruction-V (ASIR-V) and deep learning image reconstruction (DLIR) on CT radiomics feature robustness. A standardized phantom was scanned under single-energy CT (SECT) and dual-energy CT (DECT) modes at standard and low (20 and 10 mGy) dose levels. Images of SECT 120 kVp and corresponding DECT 120 kVp-like virtual monochromatic images were generated with filtered back-projection (FBP), ASIR-V at 40% (AV-40) and 100% (AV-100) blending levels, and DLIR algorithm at low (DLIR-L), medium (DLIR-M), and high (DLIR-H) strength levels. Ninety-four features were extracted via Pyradiomics. Reproducibility of features was calculated between standard and low dose levels, between reconstruction algorithms in reference to FBP images, and within scan mode, using intraclass correlation coefficient (ICC) and concordance correlation coefficient (CCC). The average percentage of features with ICC > 0.90 and CCC > 0.90 between the two dose levels was 21.28% and 20.75% in AV-40 images, and 39.90% and 35.11% in AV-100 images, respectively, and increased from 15.43 to 45.22% and from 15.43 to 44.15% with an increasing strength level of DLIR. The average percentage of features with ICC > 0.90 and CCC > 0.90 in reference to FBP images was 26.07% and 25.80% in AV-40 images, and 18.88% and 18.62% in AV-100 images, respectively, and decreased from 27.93 to 17.82% and from 27.66 to 17.29% with an increasing strength level of DLIR. DLIR and ASIR-V algorithms showed low reproducibility in reference to FBP images, while the high-strength DLIR algorithm provides an opportunity for minimizing radiomics variability due to dose reduction.

**Supplementary Information:**

The online version contains supplementary material available at 10.1007/s10278-023-00901-1.

## Introduction

Radiomics converts medical imaging data into high-dimensional minable features for constructing diagnostic, prognostic, or predictive models to aid clinical decision-making [1–6]. However, the issue of robustness should be evaluated before applying radiomics as a daily tool in clinical practice [6–10]. It is of interest to better understand the influence of acquisition and reconstruction parameters on radiomics robustness [11–19]. It has been shown that single-energy CT (SECT) and dual-energy CT (DECT) scan modes and discrepancy in technique setups among platforms impact the reproducibility of radiomic features [13–19]. Owing to developments in CT acquisition technique and reconstruction algorithms [20–22], low-dose CT has been realized with comparable and even better image quality [23–28]. Compared to the traditional filtered back projection (FBP), iterative reconstruction (IR) algorithms and deep learning image reconstruction (DLIR) algorithm have been successively introduced to provide better image quality with lower radiation dose [20–28], but their influence on the radiomics features has not been fully investigated.

The application of low-dose CT scan protocols and new reconstruction algorithms becomes a potential source of radiomics variability. It is necessary to find a way to reduce radiomics variability due to the use of scan protocols at different dose levels, and to allow the translation of radiomics models derived at high-dose level to lower ones. On one hand, CT radiomics features are sensitive to diverse reconstruction algorithms and present significant variation when comparing different strength levels [12, 29–31]. On the other hand, the difference between images acquired at distinct low and ultra-low dose levels can be bridged by careful adjusting of strength levels of IR algorithms [29–31]. Nevertheless, it is unclear how reconstruction algorithms, such as the DLIR algorithm, impact the underlying minable information in images [14]. Although the deep learning reconstruction (DLR) algorithm by Canon Medical Systems showed potential for improving radiomics reproducibility in SECT images [32], the potential of the DLIR algorithm by GE Healthcare for reducing SECT and DECT radiomics variability is still unknown.

Therefore, the aim of this study was to investigate the influence of DLIR on the robustness of radiomics features and to find out whether DLIR provided an opportunity for minimizing CT radiomics variability at different dose levels.

## Materials and Methods

### Phantom

**Fig. 1:**
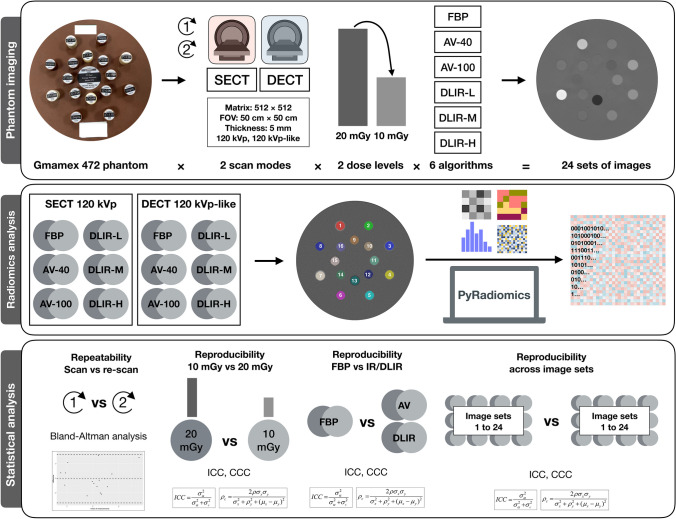
Study workflow. The current study consists of three steps, namely phantom imaging, radiomics analysis, and statistical analysis. A standardized phantom with sixteen clinical-relevant densities was scanned on a 256-slice rapid kVp-switching dual-energy CT scanner using SECT and DECT acquisition techniques, respectively, with comparable parameters at standard and low (20 and 10 mGy) dose levels. Images of SECT 120 kVp and corresponding DECT 120 kVp-like virtual monochromatic images at an energy level of 70 keV were generated. Six reconstruction algorithms were applied: FBP, adaptive statistical iterative reconstruction-V (ASIR-V, GE Healthcare) at 40% (AV-40) and 100% (AV-100) blending levels, and DLIR (TrueFidelity™, GE Healthcare) at low (DLIR-L), medium (DLIR-M) and high (DLIR-H) strength levels. Therefore, 24 sets of images were generated in total. Pyradiomics was employed to extract 19 first-order and 75 texture radiomics features from ROIs segmented with a rigid registration. The repeatability of features was assessed by Bland–Altman analysis for repeated scans. Reproducibility of features was calculated between standard and low dose levels within the same scan mode, and between reconstruction algorithms in reference to FBP images, and across 24 sets of images, using intraclass correlation coefficient (ICC) and concordance correlation coefficient (CCC). The percentage of reproducible features and ICC and CCC values were compared

The workflow of the present study is presented in Fig. 1. The ethics approval was not required because this was a phantom study. A 330-mm diameter Gammex phantom (Gammex Inc.) made of water-equivalent material was scanned. The phantom has sixteen 28-mm diameter holes for holding interchangeable inserts with various densities. To mimic contrast media in blood vessel, five iodine inserts with concentrations from 2.0 to 15.0 mg/mL were selected. To simulate a wide range of densities in the human body, 11 tissue inserts with densities of 0.44 to 1.69 g/cm^3^ were chosen. The inserts were placed to avoid beam-hardening artifacts, and their positions remained the same across the scans in the study.

### Image Acquisition and Reconstruction

All images were acquired on a 256-slice CT scanner with dual-energy CT imaging capability (Revolution Apex CT, GE Healthcare). The acquisition parameters are presented in Table 1. The SECT and DECT scans were performed with conventional 120 kVp and the rapid kVp switching dual-energy imaging technology (80/140 kVp), respectively, at two dose levels (volume CT dose indexes, CTDIVol 20 and 10 mGy). The 20 mGy dose level was selected as the reference level for an adult abdomen based on the clinical practice in our institution [33]. The 10 mGy level was selected to simulate low-dose protocol, following the previous study results indicating that using a high strength level of the DLIR algorithm could potentially reduce half of the radiation dose [23–28]. Tube currents and rotation time were modified to obtain these two dose levels. The scan field of view (500 mm × 500 mm), matrix size (512 × 512), and section thickness (5 mm) were kept the same across all scans. All the scans were repeated, several minutes after the first scan after reposition, to allow repeatability analysis.

**Table 1:** CT acquisition parameters

Scan mode	Tube voltage (kVp)	Milliamperage (mAs)	Revolution time (sec)	Pitch	Volume CT dose index (mGy)	Reconstruction kernel
SECT	120	180	0.8	0.984	10.00	Standard
SECT	120	215	0.7	0.516	19.98	Standard
DECT	80/140	335	0.6	0.984	10.00	Standard
DECT	80/140	370	1.0	0.984	19.75	Standard

The conventional 120-kVp images were generated for SECT scans. The virtual monochromatic images (VMI) were reconstructed at 70 keV to create 120-kVp-like images for DECT scans, since the CT numbers of images at the energy level of 70 keV were used as a clinical standard of reference at our institution and were reported to be comparable to those of 120-kVp images [34]. A standard reconstruction kernel was used for all reconstructions. Six reconstruction algorithms were applied: FBP, adaptive statistical iterative reconstruction-V (ASIR-V, GE Healthcare) at 40% (AV-40) and 100% (AV-100) blending levels, and DLIR (TrueFidelity™, GE Healthcare) at low (DLIR-L), medium (DLIR-M), and high (DLIR-H) strength levels. Therefore, 24 sets of images were generated in total.

### Segmentation and Feature Extraction

The circular regions of interest (ROIs) were plotted using an open-source ITK-SNAP software version 3.6.0 (http://www.itksnap.org/pmwiki/pmwiki.php). We copied the ROIs from one scan to another with rigid registrations to minimize extra variations due to segmentation. Sixteen 25-mm- (26-pixel) diameter circular ROIs were drawn at the center of each insert, covering each insert as much as possible while avoiding touching its edge. To present the true difference among platforms, we did not employ any image pre-processing steps. The radiomics features were extracted via Python version 3.7.6 (https://www.python.org) with Pyradiomics version 3.0 (https://pyradiomics.readthedocs.io/en/latest/) from each ROI on original images. Since the shape and size of ROIs were fixed, the 26 shape-based features were excluded. Within 94 extracted features, 19 were first-order features, and 75 were texture features. The details of radiomics analysis methods are presented in Supplementary Note S1.

### Radiomics Robustness Analysis

The robustness of radiomics features was evaluated in terms of repeatability and reproducibility. Test–retest repeatability of features was assessed by Bland–Altman analysis for repeated scans, with a cutoff value of 90% [35]. To test the hypothesis that the obtained biases of the radiomics feature values between the scan and re-scan were equal to zero, a one-sample *t*-test was performed. The reproducibility of radiomic features was estimated using intraclass correlation coefficient (ICC) with single rater, absolute agreement, two-way random effects model [36], and concordance correlation coefficient (CCC) [37, 38]. The reproducibility of features was calculated between the standard and low-dose levels for each reconstruction algorithm. The reproducibility of features was also estimated between reconstruction algorithms in reference to the FBP images for each scan mode per dose level, to find out whether the reconstruction algorithm alters information in images. The FBP images were used as reference, as they were considered the original version of the images. We further evaluated the reproducibility across all 24 image sets, to identify potential opportunity for minimizing radiomics variability. The reproducibility of features was considered excellent if ICC or CCC was > 0.9, good if ICC or CCC was > 0.75 and ≤ 0.9, moderate if ICC or CCC was > 0.5 and ≤ 0.75, and poor if ICC or CCC was ≤ 0.5 [39–41].

### Statistical Analysis

The statistical analysis was performed with R language version 4.1.3 (https://www.r-project.org/) within RStudio version 1.4.1106 (https://www.rstudio.com/) [42]. The proportions of reproducible radiomic features as nominal variables are presented as the percentage and were compared among different reconstruction algorithms using Cochran’s *Q* test. ICC and CCC values as continuous variables are presented as average value and were compared among different reconstruction algorithms using the Friedman test. A *P* value less than 0.05 was considered statistically significant. The significance threshold for adjusted *P* values was set at 0.05, applying the Bonferroni method for post hoc pairwise multiple-comparison correction. The details of data analysis methods are presented in Supplementary Note S2.

## Results

### Test–Retest Repeatability Analysis

The average percentages of features that met the criteria of repeatability in SECT scans and DECT scans were 91.31% and 95.04% at the 10 mGy dose level, and 90.60% and 96.81% at the 20 mGy dose level, respectively (detailed results are presented in Supplementary Fig. S1 and Supplementary Table S1). The biases of the radiomics feature values between the scan and re-scan were not significantly different from zero (all *P* > 0.05).

### Reproducibility of Radiomic Features Between Dose Levels

**Fig. 2:**
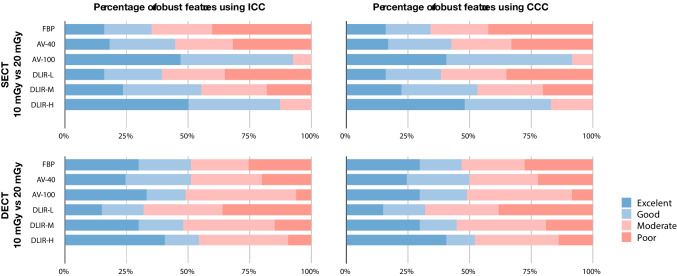
Percentage of reproducible features between dose levels. The reproducibility of features was considered excellent if ICC or CCC was > 0.9, good if ICC or CCC was > 0.75 and ≤ 0.9, moderate if ICC or CCC was > 0.5 and ≤ 0.75, and poor if ICC or CCC was ≤ 0.5

The average percentage of features with ICC > 0.90 and CCC > 0.90 was 21.28% and 20.75% for AV-40 images, and 39.90% and 35.11% for AV-100 images, respectively. Detailed results are presented in Fig. 2 and Supplementary Figs. S2 and S3. The improvements for the AV-100 images were mainly identified in the texture features. The average percentage of features with ICC > 0.90 and CCC > 0.90 between images acquired at 10 and 20 mGy dose levels increased with increasing strength level of the DLIR algorithm from 15.43 to 45.22% and from 15.43 to 44.15%, respectively, which was supported by corresponding mean ICC and CCC values. Detailed results are presented in Table 2 and Supplementary Table S2.

**Table 2:** Reproducibility of radiomic features between 10 and 20 mGy dose level images

Reconstruction algorithm	ICC > 0.90, %	ICC, mean	CCC > 0.90, %	CCC, mean
SECT (*N* = 94)				
FBP	15.96%	0.5589	15.96%	0.5489
AV-40	18.09%	0.6449	17.02%	0.6348
AV-100	46.81%	0.8746	40.43%	0.8678
DLIR-L	15.96%	0.6014	15.96%	0.5915
DLIR-M	23.40%	0.7297	22.34%	0.7199
DLIR-H	50.00%	0.8762	47.87%	0.8697
*P* value	< 0.001	< 0.001	< 0.001	< 0.001
Overall	28.37 ± 15.79%	0.7143 ± 0.1370	26.60 ± 14.00%	0.7054 ± 0.1386
DECT (*N* = 94)				
FBP	29.79%	0.7030	29.79%	0.6933
AV-40	24.47%	0.6700	24.47%	0.6613
AV-100	32.98%	0.7593	29.79%	0.7496
DLIR-L	14.89%	0.7232	14.89%	0.5602
DLIR-M	29.79%	0.7623	29.79%	0.7131
DLIR-H	40.43%	0.5709	40.43%	0.7534
*P* value	< 0.001	< 0.001	< 0.001	< 0.001
Overall	28.72 ± 8.56%	0.6981 ± 0.0714	28.19 ± 8.34%	0.6885 ± 0.0718

*P* value indicates the results of comparisons using Cochran’s *Q* test for proportions of reproducible radiomic features and the results of comparisons using Friedman test for ICC and CCC as continuous variables. The results of post hoc multiple pairwise comparisons applying the Bonferroni method for correction are presented in Supplementary Table S2

### Reproducibility of Radiomic Features in Reference to FBP Images

**Fig. 3:**
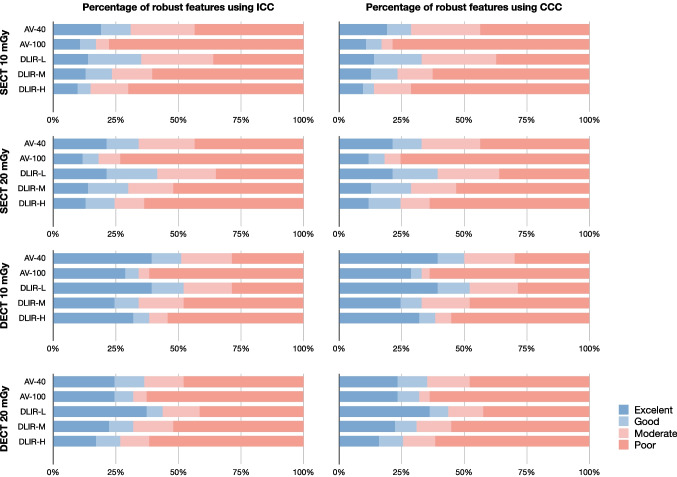
Percentage of reproducible features in reference to FBP images. The reproducibility of features was considered excellent if ICC or CCC was > 0.9, good if ICC or CCC was > 0.75 and ≤ 0.9, moderate if ICC or CCC was > 0.5 and ≤ 0.75, and poor if ICC or CCC was ≤ 0.5

The average percentage of features with ICC > 0.90 and CCC > 0.90 in reference to FBP images was 26.06% and 25.80% for AV-40 images, and 18.88% and 18.62% for AV-100 images, respectively. Detailed results are presented in Fig. 3 and Supplementary Figs. S4 and S5. The average percentage of the feature with ICC > 0.90 and CCC > 0.90 in reference to FBP images decreased with increasing strength level of the DLIR algorithm, from 27.93 to 17.82% and from 27.66 to 17.29%, respectively, which was supported by corresponding mean ICC and CCC values (Table 3 and Supplementary Table S3). In both cases, the reproducibility decreased more obviously within the texture features due to the stronger image noise reduction with the increased reconstruction strengths.

**Table 3:** Reproducibility of radiomic features in reference to FBP images

Reconstruction algorithm	ICC > 0.90, %	ICC, mean	CCC > 0.90, %	CCC, mean
SECT 10 mGy (*N* = 94)				
AV-40	19.15%	0.5427	19.15%	0.5336
AV-100	10.64%	0.2865	10.64%	0.2806
DLIR-L	13.83%	0.5747	13.83%	0.5646
DLIR-M	12.77%	0.4373	12.77%	0.4282
DLIR-H	9.57%	0.3410	9.57%	0.3334
*P* value	0.001	< 0.001	0.001	< 0.001
Overall	13.19 ± 3.73%	0.4365 ± 0.1245	13.19 ± 3.73%	0.4281 ± 0.1230
SECT 20 mGy (*N* = 94)				
AV-40	21.28%	0.5708	21.28%	0.5611
AV-100	11.70%	0.3201	11.70%	0.3135
DLIR-L	21.28%	0.6180	21.28%	0.6081
DLIR-M	13.83%	0.4978	12.77%	0.4884
DLIR-H	12.77%	0.4156	11.70%	0.4070
*P* value	< 0.001	< 0.001	< 0.001	< 0.001
Overall	16.17 ± 4.72%	0.4845 ± 0.1196	15.74 ± 5.07%	0.4756 ± 0.1183
DECT 10 mGy (*N* = 94)				
AV-40	39.36%	0.6678	39.36%	0.6605
AV-100	28.72%	0.4339	28.72%	0.4285
DLIR-L	39.36%	0.6761	39.36%	0.6686
DLIR-M	31.91%	0.5218	31.91%	0.5136
DLIR-H	24.47%	0.5043	24.47%	0.4978
*P* value	< 0.001	< 0.001	< 0.001	< 0.001
Overall	32.77 ± 6.58%	0.5608 ± 0.1067	32.77 ± 6.58%	0.5538 ± 0.1061
DECT 20 mGy (*N* = 94)				
AV-40	24.47%	0.5308	23.40%	0.5226
AV-100	24.47%	0.3932	23.40%	0.3882
DLIR-L	37.23%	0.5953	36.17%	0.5885
DLIR-M	22.34%	0.4789	22.34%	0.4711
DLIR-H	17.02%	0.4190	15.96%	0.4112
*P* value	< 0.001	< 0.001	< 0.001	< 0.001
Overall	25.11 ± 7.43%	0.4834 ± 0.0823	24.26 ± 7.35%	0.4763 ± 0.0818

*P* value indicates the results of comparisons using Cochran’s *Q* test for proportions of reproducible radiomic features and the results of comparisons using the Friedman test for ICC and CCC as continuous variables. The results of post hoc multiple pairwise comparisons applying the Bonferroni method for correction are presented in Supplementary Table S3

### Reproducibility of Radiomics Features Within Scan Mode

**Fig. 4:**
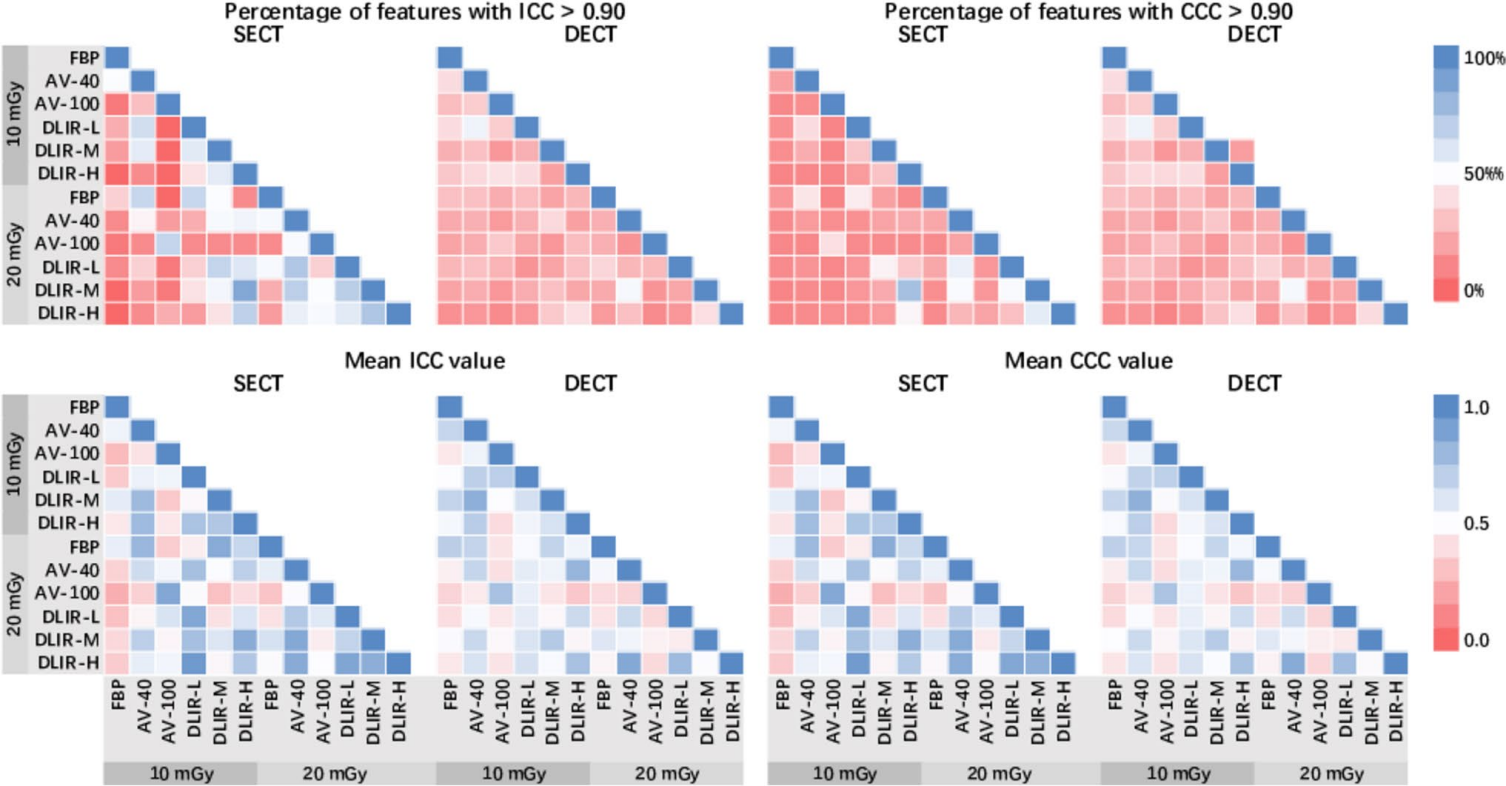
Percentage of reproducible features and mean ICC and CCC values within scan mode. Percentages indicated the features met the criteria of ICC or CCC was > 0.9. The values indicated the mean ICC or CCC values

The overall reproducibility within scan mode was low (Fig. 4). Within SECT scans at different dose levels, DLIR-H images at 10 mGy and DLIR-M images at 20 mGy showed the highest percentage with ICC > 0.90 and CCC > 0.90 of 79.78% and 76.60%. Within the same reconstruction algorithm, DLIR-H images at 10 and 20 mGy showed the highest percentage of features with ICC > 0.90 and CCC > 0.90 of 50.00% and 47.87%. The percentage of features with ICC > 0.90 and CCC > 0.90 were 46.81% and 40.43% between AV-100 images at 10 and 20 mGy. Within DECT scans at different dose levels, DLIR-H images at 10 and 20 mGy presented the highest percentage of features with ICC > 0.90 and CCC > 0.90 of 40.42% and 40.42%. The percentage of features with ICC > 0.90 and CCC > 0.90 were 32.97% and 29.79% between AV-100 images at 10 and 20 mGy.

## Discussion

In this study, we investigated the influence of ASIR-V and DLIR algorithms on the robustness of radiomics features in reference to the traditional FBP reconstruction algorithm and evaluated whether DLIR provided an opportunity for minimizing CT radiomics variability existing at different dose levels. Our study showed that the reproducibility in reference to FBP images was generally low and decreased with increasing strength level of ASIR-V and DLIR algorithms, indicating image reconstruction algorithms potentially altered radiomics features. However, the reproducibility between the standard and low dose levels increased with increasing strength level of ASIR-V and DLIR algorithms, showing the potential for minimizing radiomics variability with higher reconstruction strength for using acquisitions at different dose levels. Overall, the DLIR algorithm showed a higher possibility for reducing variability due to dose reduction than the ASIR-V algorithm. Within SECT scans, DLIR-H images at the dose levels of 10 and 20 mGy were considered the most reproducible when the same reconstruction algorithm was used. Within DECT scans, the DLIR-H algorithm showed the highest reproducibility between images at 10 and 20 mGy. These results provided insights for retrospective data collection and future protocol implementations.

The dose reduction is an important source of nonreproducible features caused by the higher image noise at reduced radiation dose levels [12, 29–31]. The higher level of ASIR-V and DLIR algorithms allow greater reduction of image noise [23–28] and are therefore expected to provide higher reproducibility of features between images acquired at different dose levels. Our results showed that the highest level of ASIR-V (AV-100) and DLIR (DLIR-H) images both had a better ability to reduce dose-induced radiomics variability within both SECT and DECT scans. We believe that ASIR-V and DLIR algorithms could at least partially harmonize the radiomics variability due to dose reduction protocols. In an era of pursuing lower radiation dose as possible, our findings may have important implications, because they provided insights into the possibility for generalizability of radiomics models derived from scan protocols of different dose levels.

However, the higher strength level of ASIR-V and DLIR algorithms may further alter radiomics features compared with the FBP algorithm. The reproducibility of AV-100 in reference to FBP images was low. The ASIR-V algorithm reduces and regulates image noise using nonlinear operations, which allows improvement of the reproducibility of features for different scans. It comes at the expense of impaired reproducibility in reference to other reconstruction algorithms such as FBP, because the ASIR-V algorithm with a higher strength level further alters the image texture from FBP when it reduces the noise [23]. The reproducibility of DLIR-H images in reference to FBP images was also low. Nevertheless, there is some doubt whether FBP images are informative enough because a significant part of the reproducible results in FBP images is considered due to repetitive noise [32]. The features which are not closely related to noise (e.g., mean) were stable among FBP, ASIR-V, and DLIR images, while those reflecting correlations between pixels (e. g. the majority of texture features) showed a decreasing trend of reproducibility with increasing strength level of ASIR-V and DLIR algorithms. DLIR algorithm uses a deep learning neural network to remove noise and is expected to maintain texture in the FBP images [23–28]. We suspected that the significant noise reduction could explain in part the low radiomics reproducibility between FBP and DLIR images. In theory, the DLIR algorithm has more possibility to preserve original informative features than the IR algorithm, because the high strength level of the DLIR algorithm did not significantly change image texture [32] and was more acceptable for clinical diagnosis than a high strength level of IR algorithm [28, 43–48]. Meanwhile, DLIR-H images did show higher reproducibility of features between images acquired at two dose levels than that of AV-100 images. Therefore, further study is recommended to investigate whether the altered radiomics features due to the high strength level of the DLIR algorithm have an impact on the discriminative power of these features.

Comparison of the DLR algorithm (Canon Medical System) with FBP and IR algorithms has shown the advantage of the DLR algorithm for improving the yield of stable and reliable radiomics features in SECT images [32]. However, DLR and DLIR trained their models with different gold standards: DLR uses model-based image reconstruction images, while DLIR uses the high-dose FBP images. Therefore, they have different behaviors in noise reduction [48]. To maximize the data usage, especially retrospectively, in clinical applications, it is of interest to explain the varying reproducibility of images acquired at different dose levels and reconstruction algorithms. Our study applied the DLIR algorithm (GE Healthcare) to confirm the potential of deep learning for reproducible CT radiomics in both SECT and DECT images and further demonstrated that the increasing strength level of the DLIR algorithm allowed higher reproducibility for CT scans of different dose levels. Our study revealed opportunities with the DLIR algorithm in retrospective data collection and future protocol implementations for radiomics [49]. The current work differed from previous studies that applied the deep learning method as an image conversion filter to improve CT radiomics reproducibility [50, 51], but underlined that image reconstruction with the deep learning method has a high potential to improve radiomics research.

Several limitations of our study should be acknowledged. First, our study was a phantom study. Therefore, the results of our study should be carefully interpreted as hypothesis generating. The generalizability of our results to tumors or diseases in clinical application may be limited, partially due to the homogeneity of our inserts [52]. However, we consider our findings to give an important insight into the performance of different reconstruction algorithms and whether the DLIR algorithm could reduce variability in radiomics features from clinical examinations. Second, we only assessed the reproducibility between standard dose and half-dose protocols. Our findings may not directly guide algorithm selection in clinical when the degree of radiation reduction varies, especially when ultra-low dose protocols are used, but we believe our study demonstrated the possibility for the DLIR algorithm to improve radiomics reproducibility even with a greater dose reduction. Third, we did not investigate the influence of reconstruction algorithms on the discriminative power of radiomics features. Because DLIR and IR algorithms may alter radiomics features in reference to FBP images, further studies are required to evaluate their impact on the reproducibility of radiomics features as diagnostic, prognostic, or predictive biomarkers [32]. Finally, our study was performed with the only available standard kernel in one CT system. Other manufacturers provide different deep learning-based algorithms with distinctly different reconstruction kernels for clinical use and may have different effects on radiomics reproducibility from those obtained in the current study [53].

To summarize, increasing the strength level of ASIR-V and DLIR algorithms improved the reproducibility of features between standard and low dose levels but decreased the reproducibility of features in reference to FBP images. DLIR algorithm may be applied for minimizing radiomics variability when combining images from protocols with different radiation doses is desired.

### Supplementary Information

Below is the link to the electronic supplementary material.

Supplementary file1 (DOCX 15077 KB)

## Data Availability

All data generated or analyzed during this study are included in this published article and its supplementary information files.

## Abbreviations

ASIR-V: Adaptive statistical iterative reconstruction-V
CCC: Concordance correlation coefficient
DECT: Dual-energy computed tomography
DLIR: Deep learning image reconstruction
DLR: Deep learning reconstruction
FBP: Filtered back-projection
ICC: Intraclass correlation coefficient
IR: Iterative reconstruction
ROI: Region of interest
SECT: Single-energy computed tomography
VMI: Virtual monoenergetic image
