# Supplementary material for: Impacts of Adaptive Statistical Iterative Reconstruction-V and Deep Learning Image Reconstruction Algorithms on Robustness of CT Radiomics Features: Opportunity for Minimizing Radiomics Variability Among Scans of Different Dose Levels
Source: J Imaging Inform Med. 2024 Jan 29;37(1):123–33. doi: 10.1007/s10278-023-00901-1 (PMC10976956; doi:10.1007/s10278-023-00901-1)
Supplement: Supplementary file 1 — Supplementary file1 (DOCX 15077 KB) [file 10278_2023_901_MOESM1_ESM.docx]

**Supplementary Materials**

**Title:** Impacts of adaptive statistical iterative reconstruction-V and deep learning image reconstruction algorithms on robustness of CT radiomics features: opportunity for minimizing radiomics variability among scans of different dose levels

**List of Supplementary Materials**

**Supplementary Notes**

Supplementary Note S1 Radiomics analysis method

Supplementary Note S2 Data analysis method

**Supplementary Tables**

Supplementary Table S1 Test-retest repeatability analysis

Supplementary Table S2 Pairwise comparison of reproducibility of radiomic features between dose levels

Supplementary Table S3 Pairwise comparison of reproducibility of radiomic features in reference to FBP images

**Supplementary Figures**

Supplementary Figure S1 Heatmap of repeatability between scan-rescan

Supplementary Figure S2 Heatmap of reproducibility of radiomic features between 10 and 20 mGy images

Supplementary Figure S3 Mean ICC and CCC values of radiomic features between 10 and 20 mGy images

Supplementary Figure S4 Heatmap of reproducibility of radiomic features in reference to FBP images

Supplementary Figure S5 Mean ICC and CCC values of radiomic features in reference to FBP images

**Supplementary Note S1 Radiomics analysis method**

**(a) ROI segmentation**

We drew the ROIs by using an open-source software ITK-SNAP software version 3.6.0 (<http://www.itksnap.org/pmwiki/pmwiki.php>), following a rigid registration to minimize variations [1-3]. We copied the ROIs from one examination to another. Sixteen ROIs (ROI 1 to 16) were circles of 25 mm (26 pixels) in diameter set at the center of each rod, to cover each rod as much as possible, and avoid to touch its edge.

The following figures illustrate the placing of 16 ROIs for corresponding inserts. Sixteen inserts were used to present multiple clinical-relevant densities, including five rods with iodine (Iod) concentrations of 2.0 mg/mL, 2.5 mg/mL, 5.0 mg/mL, 7.5 mg/mL, and 15.0 mg/mL, and eleven rods with human body densities, namely lung (0.44 g/cm^3^), adipose (0.93 g/cm^3^), breast (0.96 g/cm^3^), solid water (0.99 g/cm^3^), brain (1.04 g/ cm^3^), liver (1.06 g/cm^3^), inner bone (1.09 g/cm^3^), bone (1.10 g/cm^3^), cortical bone (CB) 2-30% (1.28 g/cm^3^), cortical bone (CB) 2-50% (1.47 g/cm^3^), and cortical bone (1.69 g/cm^3^).

**
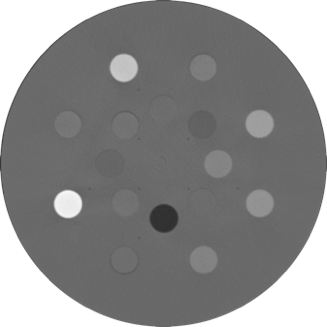
******

**(b) Feature extraction**

To present the true difference among platforms, we did not employ any image pre-processing procedure. Python version 3.7.6 (<https://www.python.org>) with Image Biomarker Standardisation Initiative (IBSI)-compliant Pyradiomics package version 3.0 (<https://pyradiomics.readthedocs.io/en/latest/>) was used to extract the radiomics features from the original images. Since the ROIs were stable, we excluded 26 shape-based features. Consequently, 94 radiomics features were extracted from each ROI, namely 19 order features and 75 texture features.

**Reference:**

1. Berenguer R, Pastor-Juan MDR, Canales-Vázquez J et al (2018) Radiomics of CT features may be nonreproducible and redundant: influence of CT acquisition parameters. Radiology 288(2):407-415
2. Chen Y, Zhong J, Wang L et al (2022) Robustness of CT radiomics features: consistency within and between single-energy CT and dual-energy CT. Eur Radiol 32(8):5480-5490
3. Zhong J, Xia Y, Chen Y et al (2023) Deep learning image reconstruction algorithm reduces image noise while alters radiomics features in dual-energy CT in comparison with conventional iterative reconstruction algorithms: a phantom study. Eur Radiol 33(2):812-824

**Supplementary Note S2 Data analysis method**

The radiomics robustness and statistical analysis was performed with R language version 4.1.3 (<https://www.r-project.org/>) within RStudio version 1.4.1106 (<https://www.rstudio.com/>) [1]. The robustness of radiomics features were evaluated in terms of repeatability and reproducibility [2,3].

**(a) Repeatability analysis of radiomics features**

For test-retest analysis, all repeated scans were involved. Radiomics features were extracted on 16 ROIs of the most representative middle three layers of images from two repeating scans with the same acquisition parameters on the same scanner. The ROIs were copied from the first scan to the second to preclude ROI variations. The repeatability was assessed by Bland-Altman analysis [4,5]. The percentage of feature scan-rescan measurements that did not exceed the 95% limits of agreement were estimated. The repeatable features were determined with cutoff values of 90%. To test the hypothesis that the obtained biases of the radiomics feature values between the scan and re-scan was equal to zero, a one-sample *t*-test was performed.

**(b) Reproducibility analysis of radiomics features**

The reproducibility of features was estimated using the intraclass correlation coefficient (ICC) with single rater, absolute agreement, two-way random effects model [6], and the concordance correlation coefficient (CCC) [7,8]. The reproducibility of features was calculated between standard and low dose levels within the same scan mode for each reconstruction algorithm, to identify the potential of IR and DLIR algorithms to minimize radiomics variability due to different dose levels. The reproducibility of features was estimated between reconstruction algorithms in reference to the FBP images for each scan mode per dose level, to find out whether reconstruction algorithm alters deep minable information hidden in images. The FBP images were used as reference, as they were considered as the original version of images, and widely accepted for clinical practice. We further evaluated the reproducibility across all twenty-four image sets, to find out potential opportunity for minimizing radiomics variability. This analysis may provide insights for bridging differences in images between different CT acquisition techniques and those due to applying dose reduction protocols.

Because the widely accepted criteria for reproducible radiomics feature have not been established, we followed the criteria used in previous studies: the reproducibility was considered as excellent if ICC or CCC was > 0.9; good if ICC or CCC was > 0.75 and ≤ 0.9; moderate if ICC or CCC was >0.5 and ≤ 0.75; and poor if ICC or CCC was ≤ 0.5 [6,9-11]. The 0.90 was selected as the threshold for reproducibility, those with ICC > 0.90 or CCC > 0.90 were considered as reproducible for further analysis [3].

The following formula illustrated the formulae used in our study, which followed Berenguer et al [2] and Chen et al [3].

**ICC formula:**


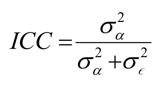


where σ^2^_α_ is the pooled variance within the subjects, and σ2ε is the trait between subjects [6].

**CCC formula:**


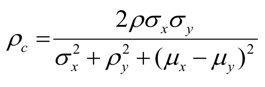


where μ_x_ and μ_y_ are the means for the 2 variables and σ^2^_x_ and σ^2^_y_ are the corresponding variances. Ρ is the correlation coefficient between the two variables [7,8].

**(c) Statistical analysis**

The proportions of reproducible radiomic features as nominal variables are presented as percentage, and were compared among different reconstruction algorithms using Cochran’s Q test. Cochran’s Q test is an extension of the McNemar test, when the response variable is dichotomous and there are either multiple times for a repeated measure or multiple categories with paired responses. A dichotomous variable is a nominal variable with only two levels. ICC and CCC values as continuous variables are presented as average value, and were compared among different reconstruction algorithms using Friedman test. The Friedman test determines if there are differences among groups for two-way data structured in a specific way, namely in an unreplicated complete block design. In this design, one variable serves as the treatment or group variable, and another variable serves as the blocking variable. We aren’t necessarily interested in differences among blocks, but we want our statistics to take into account differences in the blocks. A P value less than 0.05 was considered as statistically significant. The significance threshold for adjusted P values was set at 0.05, applying the Bonferroni method for post hoc pairwise multiple-comparison correction.

**Reference:**

1. Mangiafico SS （2016） Summary and Analysis of Extension Program Evaluation in R, version 1.19.10. Accessed via [rcompanion.org/handbook/](http://rcompanion.org/handbook/) on Apr 2022.
2. Berenguer R, Pastor-Juan MDR, Canales-Vázquez J et al (2018) Radiomics of CT features may be nonreproducible and redundant: influence of CT acquisition parameters. Radiology 288(2):407-415
3. Chen Y, Zhong J, Wang L et al (2022) Robustness of CT radiomics features: consistency within and between single-energy CT and dual-energy CT. Eur Radiol. doi: 10.1007/s00330-022-08628-3
4. Sullivan DC, Obuchowski NA, Kessler LG et al (2015) RSNA-QIBA Metrology Working Group. Metrology standards for quantitative imaging biomarkers. Radiology 277(3):813–825
5. Bland JM, Altman DG (1999) Measuring agreement in method comparison studies. Stat Methods Med Res 8(2):135–160
6. Koo TK, Li MY (2016) A Guideline of Selecting and Reporting Intraclass Correlation Coefficients for Reliability Research. J Chiropr Med 15(2):155–163
7. Lin LI (1989) A concordance correlation coefficient to evaluate reproducibility. Biometrics 45(1):255–268
8. Lin LI (2000) A note on the concordance correlation coefficient. Biometrics 56(1):324–325
9. Xue C, Yuan J, Lo GG et al (2021) Radiomics feature reliability assessed by intraclass correlation coefficient: a systematic review. Quant Imaging Med Surg 11(10):4431-4460
10. Eck B, Chirra PV, Muchhala A et al (2021) Prospective Evaluation of Repeatability and Robustness of Radiomic Descriptors in Healthy Brain Tissue Regions In Vivo Across Systematic Variations in T2-weighted magnetic resonance imaging acquisition parameters. J Magn Reson Imaging 54(3):1009-1021
11. Mitchell-Hay RN, Ahearn TS, Murray AD, Waiter GD (2022) Investigation of the inter- and intrascanner reproducibility and repeatability of radiomics features in T1-weighted brain MRI. J Magn Reson Imaging. doi: 10.1002/jmri.28191

**Supplementary Table S1 Test-retest repeatability analysis**

| Reconstruction algorithm | SECT | | DECT | | Overall |
| --- | --- | --- | --- | --- | --- |
|  | 10 mGy | 20 mGy | 10 mGy | 20 mGy |  |
| FBP | 93.62% | 88.30% | 92.55% | 96.81% | 92.82 ± 3.51% |
| AV-40 | 96.81% | 90.43% | 93.62% | 95.74% | 94.15 ± 2.81% |
| AV-100 | 89.36% | 90.43% | 94.68% | 96.81% | 92.82 ± 3.51% |
| DLIR-L | 90.43% | 92.55% | 93.62% | 93.62% | 92.55 ± 1.50% |
| DLIR-M | 87.23% | 91.49% | 98.94% | 97.87% | 93.88 ± 5.52% |
| DLIR-H | 90.43% | 90.43% | 96.81% | 100.00% | 94.41 ± 4.79% |
| Overall | 91.31 ± 3.39% | 90.60 ±1.41% | 95.04 ± 2.39% | 96.81 ± 2.13% | 93.44 ± 3.47% |

Percentages indicates the portion of feature scan-rescan measurements that did not exceed the 95% limits of agreement, when the cutoff is 0.90.

**Supplementary Table S2 Pairwise comparison of reproducibility of radiomic features between dose levels**

| Pairwise comparisons | SECT |  |  |  | DECT |  |  |  |
| --- | --- | --- | --- | --- | --- | --- | --- | --- |
|  | ICC > 0.90 | ICC mean | CCC > 0.90 | CCC mean | ICC > 0.90 | ICC mean | CCC > 0.90 | CCC mean |
| FBP vs AV-40 | > 0.999 | 0.001 | > 0.999 | < 0.001 | > 0.999 | > 0.999 | > 0.999 | > 0.999 |
| FBP vs AV-100 | < 0.001 | < 0.001 | < 0.001 | < 0.001 | > 0.999 | 0.322 | > 0.999 | 0.322 |
| FBP vs DLIR-L | > 0.999 | > 0.999 | > 0.999 | > 0.999 | 0.001 | > 0.999 | 0.001 | < 0.001 |
| FBP vs DLIR-M | > 0.999 | < 0.001 | > 0.999 | < 0.001 | > 0.999 | > 0.999 | > 0.999 | > 0.999 |
| FBP vs DLIR-H | < 0.001 | < 0.001 | < 0.001 | < 0.001 | 0.078 | > 0.999 | 0.069 | > 0.999 |
| AV-40 vs AV-100 | < 0.001 | < 0.001 | < 0.001 | < 0.001 | 0.380 | 0.018 | > 0.999 | < 0.001 |
| AV-40 vs DLIR-L | > 0.999 | 0.248 | > 0.999 | 0.290 | > 0.999 | > 0.999 | 0.162 | < 0.001 |
| AV-40 vs DLIR-M | > 0.999 | 0.003 | > 0.999 | 0.003 | > 0.999 | > 0.999 | > 0.999 | > 0.999 |
| AV-40 vs DLIR-H | < 0.001 | < 0.001 | < 0.001 | < 0.001 | < 0.001 | 0.842 | < 0.001 | 0.842 |
| AV-100 vs DLIR-L | < 0.001 | < 0.001 | < 0.001 | < 0.001 | < 0.001 | > 0.999 | 0.001 | < 0.001 |
| AV-100 vs DLIR-M | < 0.001 | 0.143 | < 0.001 | 0.101 | > 0.999 | 0.529 | > 0.999 | 0.529 |
| AV-100 vs DLIR-H | > 0.999 | > 0.999 | > 0.999 | > 0.999 | 0.756 | > 0.999 | 0.069 | > 0.999 |
| DLIR-L vs DLIR-M | < 0.001 | < 0.001 | < 0.001 | < 0.001 | 0.001 | > 0.999 | 0.001 | < 0.001 |
| DLIR-L vs DLIR-H | < 0.001 | < 0.001 | < 0.001 | < 0.001 | < 0.001 | > 0.999 | < 0.001 | < 0.001 |
| DLIR-M vs DLIR-H | > 0.999 | < 0.001 | > 0.999 | < 0.001 | 0.078 | > 0.999 | 0.069 | > 0.999 |
| Overall | < 0.001 | < 0.001 | < 0.001 | < 0.001 | < 0.001 | < 0.001 | < 0.001 | < 0.001 |

Present as adjusted P values for post hoc multiple pairwise comparisons between different reconstruction algorithms. Applying the Bonferroni method for multiple-comparison correction, the significance threshold for adjusted p values was set at 0.05, i. e., original P value * 15 = adjusted P value. Adjusted P values with statistical significance were marked in red. Left bottom indicates comparisons of ICC, right up indicates comparisons of CCC.

**Supplementary Table S3 Pairwise comparison of reproducibility of radiomic features in reference to FBP images**

| Pairwise comparisons | SECT 10 mGy |  |  |  | SECT 20 mGy |  |  |  |
| --- | --- | --- | --- | --- | --- | --- | --- | --- |
|  | ICC > 0.90 | ICC mean | CCC > 0.90 | CCC mean | ICC > 0.90 | ICC mean | CCC > 0.90 | CCC mean |
| AV-40 vs AV-100 | 0.007 | < 0.001 | 0.007 | < 0.001 | 0.002 | < 0.001 | 0.002 | < 0.001 |
| AV-40 vs DLIR-L | 0.346 | > 0.999 | 0.346 | > 0.999 | > 0.999 | 0.527 | > 0.999 | 0.527 |
| AV-40 vs DLIR-M | 0.112 | < 0.001 | 0.112 | < 0.001 | 0.037 | < 0.001 | 0.009 | < 0.001 |
| AV-40 vs DLIR-H | 0.001 | < 0.001 | 0.001 | < 0.001 | 0.009 | < 0.001 | 0.002 | < 0.001 |
| AV-100 vs DLIR-L | > 0.999 | < 0.001 | > 0.999 | < 0.001 | 0.002 | < 0.001 | 0.002 | < 0.001 |
| AV-100 vs DLIR-M | > 0.999 | < 0.001 | > 0.999 | < 0.001 | > 0.999 | < 0.001 | > 0.999 | < 0.001 |
| AV-100 vs DLIR-H | > 0.999 | > 0.999 | > 0.999 | > 0.999 | > 0.999 | 0.302 | > 0.999 | 0.302 |
| DLIR-L vs DLIR-M | 0.910 | < 0.001 | 0.910 | < 0.001 | 0.037 | < 0.001 | 0.009 | < 0.001 |
| DLIR-L vs DLIR-H | > 0.999 | < 0.001 | > 0.999 | < 0.001 | 0.009 | < 0.001 | 0.002 | < 0.001 |
| DLIR-M vs DLIR-H | > 0.999 | < 0.001 | > 0.999 | < 0.001 | > 0.999 | < 0.001 | > 0.999 | < 0.001 |
| Overall | 0.001 | < 0.001 | 0.001 | < 0.001 | < 0.001 | < 0.001 | < 0.001 | < 0.001 |
| Pairwise comparisons | DECT 10 mGy |  |  |  | DECT 20 mGy |  |  |  |
|  | ICC > 0.90 | ICC mean | CCC > 0.90 | CCC mean | ICC > 0.90 | ICC mean | CCC > 0.90 | CCC mean |
| AV-40 vs AV-100 | 0.026 | < 0.001 | 0.026 | < 0.001 | > 0.999 | < 0.001 | > 0.999 | < 0.001 |
| AV-40 vs DLIR-L | > 0.999 | > 0.999 | > 0.999 | > 0.999 | 0.001 | > 0.999 | 0.002 | > 0.999 |
| AV-40 vs DLIR-M | < 0.001 | < 0.001 | < 0.001 | < 0.001 | > 0.999 | 0.005 | > 0.999 | 0.005 |
| AV-40 vs DLIR-H | 0.348 | < 0.001 | 0.348 | < 0.001 | 0.269 | < 0.001 | 0.284 | < 0.001 |
| AV-100 vs DLIR-L | 0.026 | < 0.001 | 0.026 | < 0.001 | 0.001 | < 0.001 | 0.002 | < 0.001 |
| AV-100 vs DLIR-M | > 0.999 | 0.004 | > 0.999 | 0.004 | > 0.999 | 0.011 | > 0.999 | 0.011 |
| AV-100 vs DLIR-H | > 0.999 | 0.065 | > 0.999 | 0.065 | 0.269 | > 0.999 | 0.284 | > 0.999 |
| DLIR-L vs DLIR-M | < 0.001 | < 0.001 | < 0.001 | < 0.001 | < 0.001 | 0.003 | < 0.001 | 0.003 |
| DLIR-L vs DLIR-H | 0.348 | < 0.001 | 0.348 | < 0.001 | < 0.001 | < 0.001 | < 0.001 | < 0.001 |
| DLIR-M vs DLIR-H | 0.348 | > 0.999 | 0.348 | > 0.999 | > 0.999 | < 0.001 | 0.603 | < 0.001 |
| Overall | < 0.001 | < 0.001 | < 0.001 | < 0.001 | < 0.001 | < 0.001 | < 0.001 | < 0.001 |

Present as adjusted P values for post hoc multiple pairwise comparisons between different reconstruction algorithms. Applying the Bonferroni method for multiple-comparison correction, the significance threshold for adjusted p values was set at 0.05, i. e., original P value * 10 = adjusted P value. Adjusted P values with statistical significance were marked in red. Left bottom indicates comparisons of ICC, right up indicates comparisons of CCC.

**Supplementary Figure S1 Heatmap of repeatability between scan-rescan**

**Supplementary Figure S2 Heatmap of reproducibility of radiomic features between 10 and 20 mGy images**

**Supplementary Figure S3 Mean ICC and CCC values of radiomic features between 10 and 20 mGy images**

**Supplementary Figure S4 Heatmap of reproducibility of radiomic features in reference to FBP images**

**Supplementary Figure S5 Mean ICC and CCC values of radiomic features in reference to FBP images**
